# Signaling pathways governing breast cancer stem cells behavior

**DOI:** 10.1186/s13287-021-02321-w

**Published:** 2021-04-16

**Authors:** Kai Song, Maryam Farzaneh

**Affiliations:** 1 Xuzhou Vocational College of Bioengineering, Xuzhou, 221006 Jiangsu China; 2 Fertility, Infertility and Perinatology Research Center, Ahvaz Jundishapur University of Medical Sciences, Ahvaz, Iran

**Keywords:** Breast cancer, Breast cancer stem cells, Signaling pathways, miRNAs, Metastasis, Tumorigenesis

## Abstract

Breast cancer is the second common cancer and the leading cause of malignancy among females overall. Breast cancer stem cells (BCSCs) are a small population of breast cancer cells that play a critical role in the metastasis of breast cancer to other organs in the body. BCSCs have both self-renewal and differentiation capacities, which are thought to contribute to the aggressiveness of metastatic lesions. Therefore, targeting BCSCs can be a suitable approach for the treatment and metastasis of breast cancer. Growing evidence has indicated that the Wnt, NFκB, Notch, BMP2, STAT3, and hedgehog (Hh) signaling pathways govern epithelial-to-mesenchymal transition (EMT) activation, growth, and tumorigenesis of BCSCs in the primary regions. miRNAs as the central regulatory molecules also play critical roles in BCSC self-renewal, metastasis, and drug resistance. Hence, targeting these pathways might be a novel therapeutic approach for breast cancer diagnosis and therapy. This review discusses known signaling mechanisms involved in the stimulation or prevention of BCSC self-renewal, metastasis, and tumorigenesis.

## Introduction

Breast cancer (BC) is the most invasive cancer and the second common cause of malignancy among females overall [1–3]. BC involves different areas of the breast (lobules, ducts, and connective tissue) and shows various physiological properties and different clinical outcomes [4, 5]. Based on the cancer response, BC can be divided into estrogen receptor (ER)-positive (response to estrogen signaling), progesterone receptor (PR)-positive (response to progesterone), ER/PR-positive, and human epidermal growth factor receptor-2 (HER2)-positive tumors [6–8]. Triple-negative breast cancer (TNBC) as a subtype of basal-like breast cancer (BLBC) is defined with negative expression of the ER, PR, and HER2 [9]. The main treatments for BC include chemotherapy [10, 11], radiation therapy [12, 13], hormone-blocking therapy [14, 15], surgery [16], and biological treatment [17]. Despite available interventions, these strategies may not always be the best treatment options for targeting BC metastasis [18]. Therefore, a better understanding of the molecular mechanisms involved in tumorigenesis of BC is required to develop more effective therapeutic strategies [19, 20]. Breast cancer stem cells (BCSCs) are a small population of BC cells that play a critical role in the metastasis of BC to other organs in the body [21]. BCSCs have the ability to self-renew and to differentiate into specialized cells that are found in malignancy [22, 23]. Accumulating evidence shows that BCSCs are the leading cause of tumor progression and resistance against conventional therapy [24–26]. Therefore, targeting BCSCs may be an appropriate approach for the treatment of BC [27–30]. This review discusses known signaling mechanisms involved in the stimulation or prevention of BCSC self-renewal, metastasis, and tumorigenesis.

## Cellular and molecular characteristics of BCSCs

**Fig. 1:**
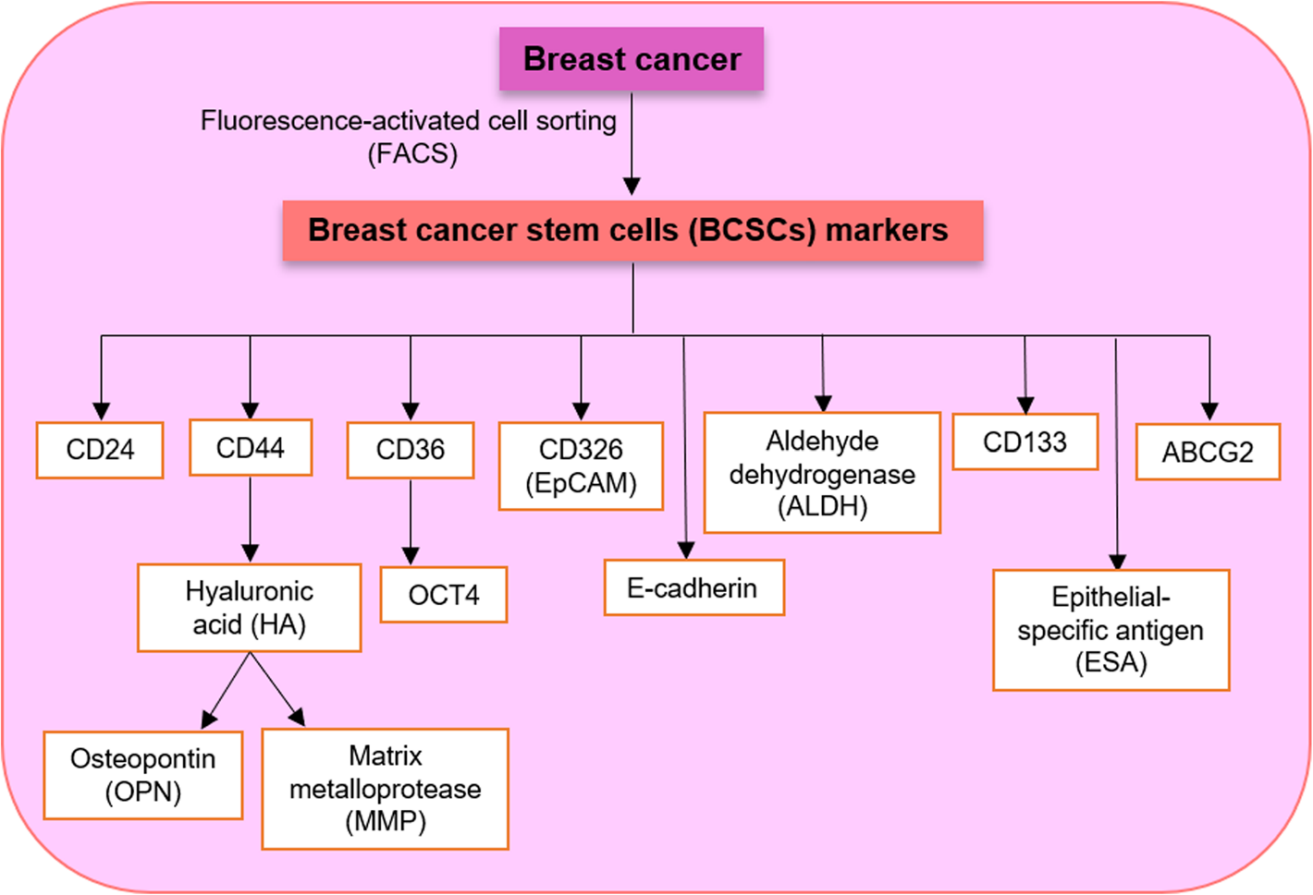
Cellular and molecular characteristics of breast cancer stem cells (BCSCs)

In recent years, the existence of BCSCs or breast cancer-initiating cells (BCICs) in BC has been confirmed [31–33]. BCSCs as a subset of cancer cells exhibit similar properties with normal stem cells [34]. These cells have a slow cell cycle and the potential to divide asymmetrically and to seed tumors when transplanted into a host [35, 36]. BCSCs have antioxidative, tumorsphere formation, tumorigenicity, and chemoresistance properties [37]. Based on cell surface marker expression (by fluorescence-activated cell sorting (FACS)), BCSCs are CD44(+)/CD24(−/low) tumorigenic cells that initiate tumors in xenografts [34, 38]. CD44 is a cell surface glycoprotein and stemness marker in BCSCs [39]. CD44 binds to hyaluronic acid (HA) and mediates the interactions between cell/cell and cell/matrix proteins such as matrix metalloprotease (MMP) and osteopontin (OPN) [37, 40]. Therefore, the HA hydrogel might be an efficient strategy that targets BCSCs [37]. CD24 is a glycosylated cell surface protein that negatively controls the function of CXCR4 (chemokine receptor) and regulates BCSC metastasis and proliferation [18, 41]. Gene expression of embryonic stem cell factors, including Oct4, Nanog, SOX2, and DNA (cytosine-5)-methyltransferase 1 (DNMT1), is observed in BCSCs [36]. It was validated that BCSCs express CD326 (EpCAM), aldehyde dehydrogenase (ALDH), epithelial-specific antigen (ESA), and E-cadherin [42, 43]. The ALDH1 enzyme is a useful therapeutic target that regulates BCSC functions and malignancies [44, 45]. EpCAM by Wnt signaling can stimulate cell adhesion, proliferation, and invasion of BCSCs [46]. CD36 through uptaking fatty acids and induction of STAT3 and nuclear factor kappa B (NFkB) can promote expression of the stem cell marker OCT4, metastasis, and migration of BCSCs [47, 48]. Histone deacetylases (HDACs) such as HDAC1 and HDAC7 play essential roles in BCSC maintenance [49]. CD47, CD133, CD166, CD61, ABCG2, and Lgr5 are the other markers used for the isolation of BCSCs [50] (Fig. 1).

There is a high degree of intertumor and intratumor heterogeneity in breast cancer [51, 52]. Thus, a single tumor may contain BCSCs with distinct molecular profiles [53, 54]. Based on immunohistochemical analyses, cells with the CD44+CD24−/low phenotype are not enough to characterize BCSC. Several candidate markers such as the ESA antigen, ALDH1 expression, Prominin-1 (CD133), and CD131 and the capacity to form spheroid can be independent factors for the characterization of BCSCs [49, 55, 56].

## Critical signaling pathways involved in the stimulation or prevention of BCSC propagation and metastasis

**Fig. 2:**
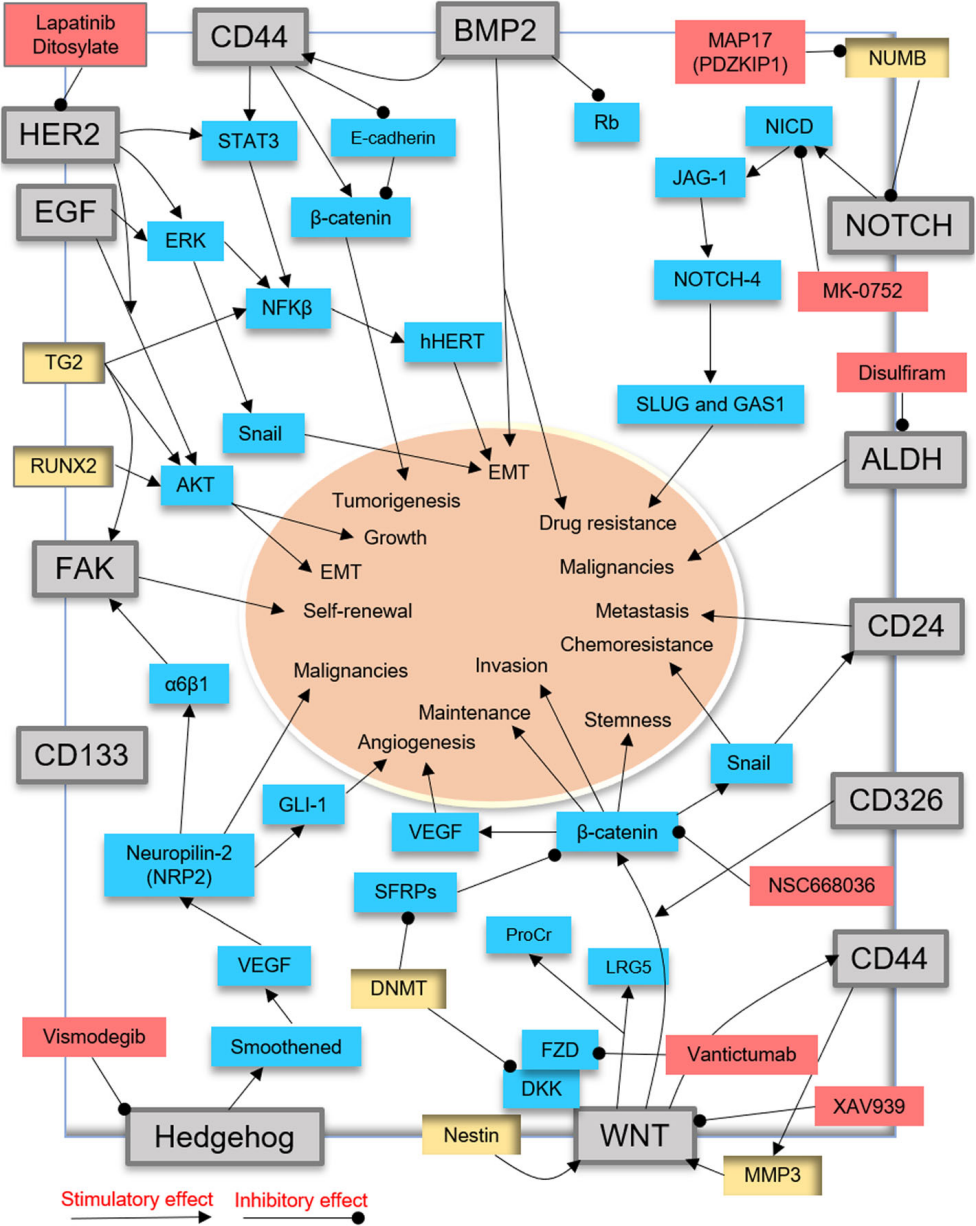
Critical signaling pathways involved in breast cancer stem cell (BCSC) propagation and metastasis

Tumor microenvironment and signaling pathways have critical roles in the propagation and differentiation of BCSCs [57, 58] (Fig. 2). Growing evidences have indicated that the Wnt/β-catenin, NFκB, BMP2, Notch, STAT3, and hedgehog (Hh) signaling pathways govern epithelial-to-mesenchymal transition (EMT) activation, growth, and tumorigenesis of BCSCs in the primary regions [38, 59–62]. However, many of these crucial signaling pathways play important functional roles in normal stem cells [63, 64]. Several specific molecules, including NFkB, BCL6, SOX2, FOXC2, and hypoxia-inducible factor-1 (HIF1), have been contributed to BCSC EMT and malignancies [39]. Recent studies showed that the Wnt-, STAT3-, HDAC-, and estrogen receptor alpha (ESR1)-related pathways can cause TNBC-associated BCSCs (TNBCSC) to undergo unexpected differentiation, EMT, and metastasis [49, 65].

### Signaling pathways induced by CD44 in BCSCs

CD44 is known to cooperate with the receptor tyrosine kinase (RTK) and regulate BCSC proliferation and migration [39]. Blockade of CD44 impairs the properties of BCSCs, including cell adhesion, malignancy, progression, metastasis, EMT, and therapy resistance [39, 66]. Several signaling pathways such as Wnt/β-catenin, PI3K/Akt, Ras-MAPK, and Rho GTPases are stimulated by CD44 [67, 68]. Thus, CD44 may be a predictor biomarker for BCSC isolation and therapy resistance [69]. However, CD44 is not a suitable marker for the detection of luminal BCSCs (CD44−/CD24+ or CD44−/CD24−) [39, 70]. CD44 can suppress the formation of the E-cadherin/β-catenin complex and enhance nuclear β-catenin and genes related to cell invasion and tumorigenesis in BCSCs [71]. CD44 also interacts with STAT3 and NF-kB to activate the catalytic subunit of telomerase (hTERT), enhance metastasis, and trigger the EMT process in BCSCs [69, 72, 73].

### WNT signaling

The Wnt pathway plays a pivotal role in BCSC phenotype shaping, proliferation, migration, chemoresistance, and radioresistance [62, 74]. Canonical and noncanonical Wnt signaling by targeting CD44 promotes the “stemness” of BCSCs [75, 76]. The Wnt/β-catenin/TCF4 axis through the Snail protein promotes the expression of miR-125b and chemoresistance in BCSCs [77]. Snail enhances the expression of CD44 marker and ALDH activity in BCSCs [78]. Protein C receptor (ProCr) and LRG5 are novel Wnt targets and potent biomarkers of BCSCs [79, 80]. MMP3 has been proven that targets Wnt signaling and contributes to the maintenance of BCSCs [81]. Nestin is a type VI intermediate filament protein that positively targets the Wnt/β-catenin pathway and enhances the metastatic ability of BCSCs [82]. DNMT has been proposed to inactivate the cytoplasmic β-catenin antagonists such as secreted frizzled-related proteins (SFRPs) and DICKKOPF protein (DKK) and to promote the expression of Wnt/β-catenin signaling in BCSCs [83, 84]. Therefore, targeting Wnt/β-catenin signaling may be a potent marker for removing BCSCs [18, 71]. Recent studies showed that NSC668036 targeted the PDZ domain and suppressed Disheveled (Dvl) and FZD interactions in Wnt/β-catenin signaling (pre-clinical trial) [85]. OMP-18R5 (Vantictumab) is a monoclonal antibody against FZD receptors that targets FZDs and blocks BCSC growth (phase I) [86]. XAV939 has been demonstrated that interacts with the type 1 and 2 tankyrase-binding domain (TBD) of the Axin molecule and blocks Wnt/β-catenin signaling in BCSCs (phase I) [85]. PKF118-310 (PKF) is a small molecule inhibitor of Wnt/β-catenin signaling that targets BCSCs in a HER2-overexpressing mouse model [87]. Pyrvinium pamoate (PP) is an anti-helminthic drug and a WNT pathway suppressor that inhibits the expression of the NANOG, SOX2, and OCT4 genes, and the growth of BCSCs [88].

### BMP2 signaling

In breast cancer xenograft models, BMP-2 can promote EMT transition and bone metastasis [89]. BMP2 via targeting CD44 expression and suppressing the Rb signaling pathway induces EMT, stemness, and chemoresistance in BCSCs [89]. Activation of the PI3K/Akt pathway as well as Rb interaction with CD44 has been shown to play essential roles in BMP-2-dependent EMT in BCSCs [89]. However, BMPs are able to cause G1 arrest, increase apoptosis, and suppress BCSC proliferation [90]. Therefore, the BMP family may have dual behaviors in stimulation or suppression of BCSCs [91]. Thus, employing BMP family inhibitors may be useful for targeting BCSCs [49, 92, 93].

### Hedgehog signaling

Hedgehog signaling by interaction with the Smoothened (SMO) protein can influence BCSC stemness and malignancies [49, 94]. Neuropilin-2 (NRP2) is a VEGF receptor that stimulates the expression of glioma-associated oncogene-1 (GLI-1) and α6β1 integrins and contributes to BCSC initiation [95]. GLI-1 by promoting angiogenesis accelerates BCSC progression [96]. Studies suggest that α6β1 integrins can trigger focal adhesion kinase (FAK) signaling and mediate BCSC self-renewal ability [97]. Therefore, targeting the VEGF/NRP2, α6β1, GLI1, and FAK signaling pathways can provide an attractive strategy for BC treatment [98]. Genistein is an isoflavone component present in soy products that has been shown to suppress hedgehog downstream signaling and block BCSC growth and survival [99]. Besides, aqueous extract of Trametes robiniophila Murr (Huaier) by blocking hedgehog downstream signaling can decrease BCSC growth, self-renewal, and proliferation [100, 101].

### Notch signaling

The Notch pathway through JAG-1 and NOTCH-4 can stimulate and maintain the invasion, mesenchymal-like properties, and drug resistance of BCSCs [102, 103]. NOTCH4 by targeting SLUG and GAS1 is involved in BC development [104]. In normal cells, the NUMB protein blocks the Notch intracellular domain (NICD) in the cytoplasm and inhibits the Notch pathway. miR-146a has been reported to suppress the function of NUMB, activate the Notch pathway, and trigger the formation of BCSCs [105]. Thus, downregulation of miR-146a and miR-146b expression may weaken the capacity for self-renewal in BCSCs [106]. MAP 17 (PDZKIP1) is a small cargo protein that negatively regulates the NUMB activity, activates the Notch pathway, and promotes the maintenance of BCSCs [107]. 6-Shogaol as a ginger-derived compound by targeting the expression of the Notch-Hes1-Cyclin D1 (CYLD) axis can suppress autophagy and apoptosis and then blocks the growth of BCSCs [108]. MK-0752 is a gamma-secretase inhibitor that inhibits the NICD domain and targets the BCSC population (phases I and II) [109]. Vismodegib (GDC-0449) is a Notch/hedgehog inhibitor drug that inhibits BCSC growth in tamoxifen-resistant breast cancer (phase I) [110] (http://clinicaltrials.gov).

### PI3K-AKT signaling

Epidermal growth factor receptor (EGFR/HER)-related signaling have been implicated in the pathogenesis of BCSCs and resistance to chemotherapeutic drugs [111, 112]. This signaling activates molecules such as STAT3, protein kinase B (PKB or AKT), and tyrosine kinase Src and stimulates the MAPK (Ras/Raf/Mek/Ek), PI3K/Akt, and STATs pathways [35]. PI3K-AKT signaling is required for BCSC phenotype, EMT, and drug resistance [113]. The role of PI3K/Akt in BCSCs may be mediated by HER2 [114]. HER1- and HER2-positive BCSCs are able to self-renew [115, 116]. HER2 dysregulation leads to a rise in the phosphorylation of Akt in the ALDH+ population of BCSCs [35]. Therefore, the effects of HER2 signaling in BCSCs can be increased through the PI3K/Akt pathway [35]. Transglutaminase (TG2) has been shown that stimulates NFkβ, Akt, and FAK signaling and initiates BCSC growth and survival [117]. Tbox transcription factor 3 (Tbx3) through FGF signaling is associated with BCSC phenotypes and oncogenesis [118, 119]. In addition, the ZFHX3 transcription factor by enhancing TBX3 transcription increases the proliferation and tumor growth of BCSCs [120]. A recent study indicated that Runt-related transcription factor 2 (RUNX2) by activating the PI3K/AKT pathway contributes to tumorigenicity, metastasis, and EMT in BCSCs [121]. It has been reported that Disulfiram (DS) as an anti-alcoholism drug can inhibit NFκB activation, inhibit ALDH enzymatic activity, and reduce BCSC stemness and chemoresistance [122]. Everolimus (RAD001) is a PI3K/Akt/mTOR pathway inhibitor that blocks BCSC growth [123, 124]. Lapatinib ditosylate (NCT01868503) by targeting EGFR/HER2 impacts on the subpopulation of BCSCs (phase II) [125] (http://clinicaltrials.gov/).

## Signaling pathways induced by miRNAs in BCSCs

Recently, studies have been suggested that epigenetic changes such as DNA methylation and histone modifications enhance the events of BCSC metastasis [126–128]. miRNAs are epigenetic modulators that target mRNAs without modifying the gene sequences [129, 130]. In human cancer, miRNA expression can be controlled by epigenetic modifications [131]. miRNAs also play an important role in the proliferation, migration, and invasion of BCSCs [132–135]. The expression of microRNAs can be deregulated in BCSCs [130]. Several miRNAs including mir-21, mir-22, mir-29a, and mir-221/222 increase tumorigenesis, and miR-34a, miR-628, miRNA-140-5p, and miRNA-4319 decrease metastasis in BCSCs [39, 136, 137].

### Stimulation of tumorigenesis

**Fig. 3:**
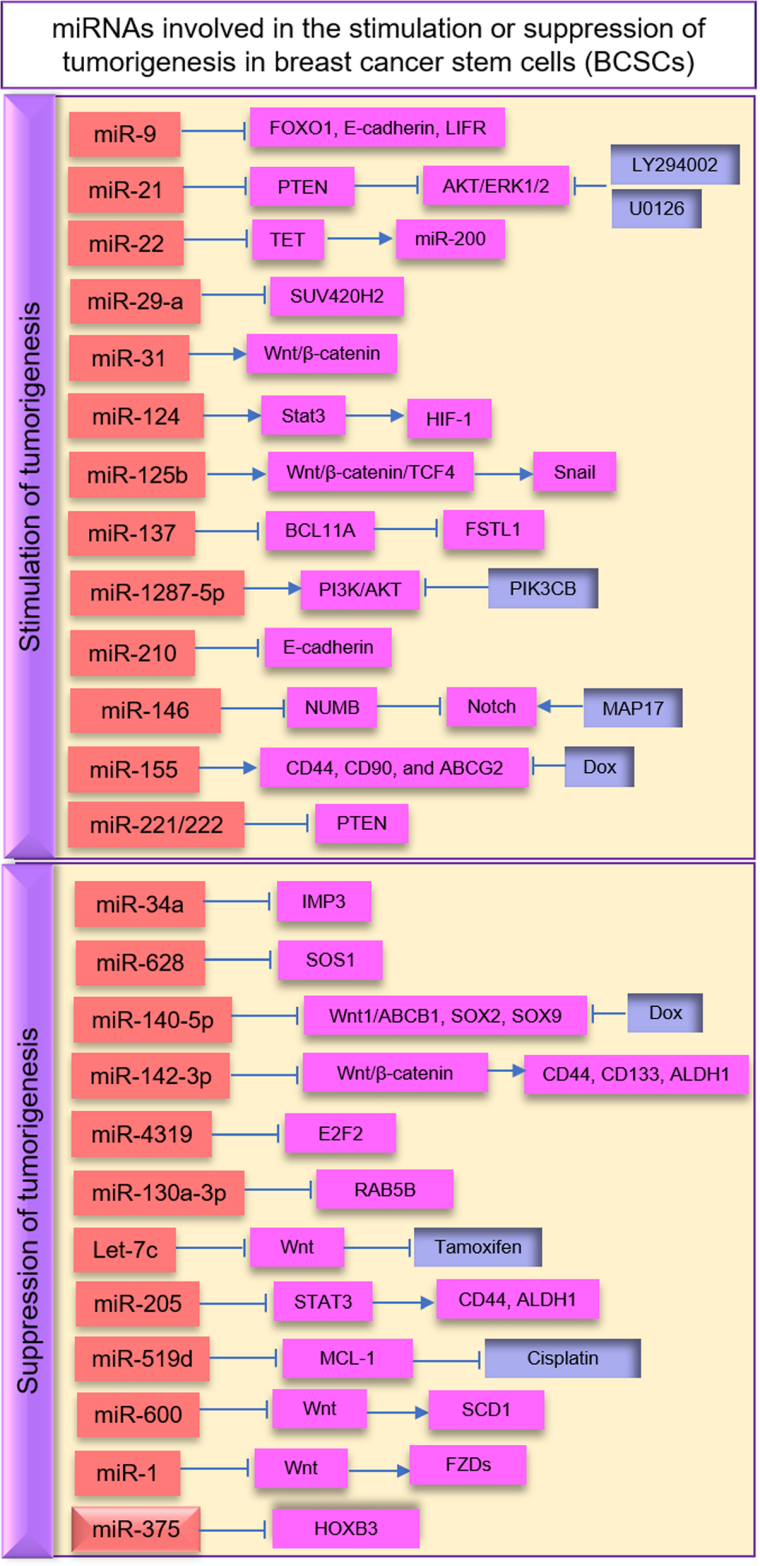
miRNAs involved in the stimulation or suppression of tumorigenesis in breast cancer stem cells (BCSCs)

miRNAs as the central regulatory molecules serve critical roles in BCSC self-renewal, metastasis, and drug resistance [138–140] (Fig. 3). miR-21 by targeting the phosphatase and tensin homolog (PTEN) protein stimulates AKT/ERK1/2 signaling and contributes to the BCSC progression, EMT, and metastasis [113]. LY294002 and U0126 as the inhibitors of the PI3K-AKT and ERK1/2 pathways suppress EMT and BCSC phenotype [113]. miR-22 has been shown to target the TET (ten eleven translocation) family of methylcytosine dioxygenases and inhibit demethylation of the miR-200 promoter, promote EMT, BCSC stemness, and metastasis [141]. miR-31 targets Wnt/β-catenin signaling and increases BCSC stemness and tumorigenesis [142]. It has been evident that miR-29a represses SUV420H2 (a histone methyltransferase) and promotes EMT progress, migration, and metastasis in BCSCs [143]. miR-124 through targeting STAT3 regulates the HIF-1 pathway and enhances doxorubicin (DOX) resistance of BCSCs [144]. miR-125b has been suggested that targets the Snail protein and increases the CD44+ and chemoresistance BCSCs [77]. miR-1287-5p through PI3K/AKT signaling plays critical roles in the prognosis and survival of BCSCs [145]. PIK3CB is a PI3Kinase pathway chemical inhibitor that interacts with miR-1287-5p and suppresses breast carcinogenesis [145]. It has been validated that miR-137 via targeting BCL11A (a zinc-finger transcription factor) and Wnt signaling enhances FSTL1 levels and chemoresistance in BCSCs [146]. The hypoxic microenvironment around BCSCs can induce the expression of miR-210. Hypoxia-mediated miR-210 by targeting E-cadherin improves BCSC invasion and proliferation [147]. miR-155 enhances BCSC stemness markers, including CD44, CD90, and ABCG2. Thus, downregulation of miR-155 promotes DOX sensitivity in BCSC [148]. It has been shown that miR-9 and miR-221 via targeting multiple genes involved with carcinogenesis are able to promote BCSC stemness and the capacity for tumor cell renewal [149]. miR-9 by targeting forkhead box O1 (FOXO1), E-cadherin, and leukemia inhibitory factor receptor (LIFR) promotes the BCSC recurrence and invasiveness [150]. miR-221/222 has been reported to regulate the expression of PTEN and enhance the growth and maintenance of BCSC [151]. Some findings suggest that miR-146a and miR-146b by targeting the Notch pathway are involved in the development of BCSCs [105].

### Suppression of tumorigenesis

Some miRNAs may act as tumor suppressors and overcome tumorigenesis and drug resistance in BSCS [138, 152]. miR-34a is an important miRNA that targets the insulin-like growth factor II (IGFII), mRNA-binding protein (IMP3)-induced stemness, and Wnt/β-catenin signaling and decreases BCSC self-renewal [153]. miR-628 by targeting SOS Ras/Rac guanine nucleotide exchange factor 1 (SOS1) inhibits BCSC migration and invasion [154]. miR-140-5p as a critical tumor suppressor blocks the Wnt/β-catenin, SOX2, and SOX9 pathways and inhibits the growth, tumorsphere formation, and progression of BCSCs [155, 156]. This miRNA through the Wnt1/ABCB1 pathway promotes the sensitivity of BCSCs to Dox [156]. miR-142-3p by targeting β-catenin pathway can reduce CD44, CD133, ALDH1, and radioresistance in BCSCs [157]. miR-4319 can suppress the expression of the E2F2 transcription factor and decrease the tumorigenicity of TNBCSC [158]. Another investigation shows that miR-130a-3p by targeting the expression of RAB5B (member of RAS oncogene family) inhibits the carcinogenic features of BCSCs [159, 160]. miRNA Let-7 has been shown to block the Wnt pathway, inhibit the growth and stemness of BCSCs, and promote the anti-cancer effect of tamoxifen (a chemotherapeutic drug) [161]. Recent work has also identified Let-7c through estrogen-activated Wnt signaling can suppress the self-renewal abilities of BCSCs [162]. miR-205 via modulating STAT3 signaling reduces the expression of CD44 and ALDH1 stem-cell markers and inhibits BCSC migration and invasion [163]. miR-519d by targeting MCL-1 (a member of the proapoptotic Bcl-2 family) increases the sensitivity of BCSC to cisplatin (a chemotherapeutic drug) [164]. miR-600 through the Wnt pathway targets stearoyl desaturase 1 (SCD1) and reduces BCSC self-renewal and tumorigenicity [165]. Also, miRNA-1 has been identified that targets frizzled receptors (FZDs) in the Wnt pathway and decreases BCSC proliferation and metastasis [166]. miR-375 by degrading the HOXB3 gene reduces BCSC phenotypes, EMT, and tamoxifen resistance [167]. Therefore, tumor-suppressing miRNAs with their functional pathways could be introduced as an effective strategy for targeting BCSCs [168, 169].

## Conclusion

Several signal transduction pathways, including Wnt/β-catenin, hedgehog, Notch, BMPs, and PI3K/Akt/NFkB, are deregulated in BCSCs. These signaling pathways stimulate proliferation, migration, invasion, EMT, chemotherapy, and radiotherapy resistance in BCSCs. miRNAs also through several signaling pathways can regulate the stemness features and tumorigenesis of BCSCs. Inhibition of key signaling pathways with small molecule inhibitors, nanoparticles, herbal medicine, and genetic modifications might be effective therapeutic approaches for targeting BCSCs [31, 85, 170, 171].

## Data Availability

The datasets used and/or analyzed during the current study are available from the corresponding authors on reasonable request.

## Abbreviations

ALDH: Aldehyde dehydrogenase
BC: Breast cancer
BCICs: Breast cancer-initiating cells
BCSCs: Breast cancer stem cells
BLBC: Basal-like breast cancer
DKK: DICKKOPF protein
Dox: Doxorubicin
DNMT: DNA methyltransferase
ESA: Epithelial-specific antigen
EMT: Epithelial-to-mesenchymal transition
EGFRs: Epidermal growth factor receptors
ER: Estrogen receptor
FAK: Focal adhesion kinase
FACS: Fluorescence-activated cell sorting
GLI-1: Glioma-associated oncogene-1
HA: Hyaluronic acid
HER2: Human epidermal growth factor receptor-2
Hh: Hedgehog
hTERT: Human telomerase
IGFII: Insulin-like growth factor II
IMP3: IGFII mRNA-binding protein
MMP3: Matrix metalloproteinase 3
NFkB: Nuclear factor kappa B
NRP2: Neuropilin-2
OCT4: Transcription factor 4
OPN: Osteopontin
PKB: Protein kinase B
PR: Progesterone receptor
ProCr: Protein C receptor
PTEN: Phosphatase and tensin homolog
RUNX2: Runt-related transcription factor 2
RTK: Receptor tyrosine kinase
SCD1: Stearoyl desaturase 1
SFRPs: Secreted frizzled-related proteins
SOS1: SOS Ras/Rac guanine nucleotide exchange factor 1
SMO: Smoothened
Tbx3: Tbox transcription factor 3
TG2: Transglutaminase
TET: Ten eleven translocations
TNBCSC: TNBC-associated BCSCs
